# Neuroprotective Peptides in Retinal Disease

**DOI:** 10.3390/jcm8081146

**Published:** 2019-08-01

**Authors:** Davide Cervia, Elisabetta Catalani, Giovanni Casini

**Affiliations:** 1 Department for Innovation in Biological, Agro-food and Forest systems (DIBAF), Università degli Studi della Tuscia, largo dell Università snc, 01100 Viterbo, Italy; 2 Department of Biology, Università degli Studi di Pisa, via San Zeno 31, 56127 Pisa, Italy; 3 Interdepartmental Research Center Nutrafood “Nutraceuticals and Food for Health”, Università degli Studi di Pisa, via del Borghetto 80, 56124 Pisa, Italy

**Keywords:** neuropeptides, receptors, vertebrate retina, retinal neurons, cell death, neuroprotection, retina neurodegeneration, retinal vessels

## Abstract

In the pathogenesis of many disorders, neuronal death plays a key role. It is now assumed that neurodegeneration is caused by multiple and somewhat converging/overlapping death mechanisms, and that neurons are sensitive to unique death styles. In this respect, major advances in the knowledge of different types, mechanisms, and roles of neurodegeneration are crucial to restore the neuronal functions involved in neuroprotection. Several novel concepts have emerged recently, suggesting that the modulation of the neuropeptide system may provide an entirely new set of pharmacological approaches. Neuropeptides and their receptors are expressed widely in mammalian retinas, where they exert neuromodulatory functions including the processing of visual information. In multiple models of retinal diseases, different peptidergic substances play neuroprotective actions. Herein, we describe the novel advances on the protective roles of neuropeptides in the retina. In particular, we focus on the mechanisms by which peptides affect neuronal death/survival and the vascular lesions commonly associated with retinal neurodegenerative pathologies. The goal is to highlight the therapeutic potential of neuropeptide systems as neuroprotectants in retinal diseases.

## 1. Introduction

Programmed neuronal cell death plays a crucial role during development because of the limited ability of adult neurons to proliferate or be replaced, while neuronal cell death may also occur in the mature nervous system because of trauma or in the presence of a neurodegenerative disease. Neuronal cell damage triggers a chain of events that may lead to DNA fragmentation, engulfment of the cell, apoptosis, autophagy, necrosis, or other types of cell death mechanisms [1]. However, the distinction between the initiating factor that induces death and the executioner mechanism is not always clear. As excellently reviewed by Brown and colleagues [2], there are many ways for neurons, which cross-talk with each other, to die, and death is often triggered by interactions with neighboring cells, including glial cells. Of interest, neurons undergo most of the common forms of cell death experienced by non-neuronal cells, although their complexity makes them sensitive/susceptible to unique death styles, including ischemia-induced death, excitotoxicity (initiated by excitatory amino acid neurotransmitters), sodium overload and swelling, calcium overload, axon rupture, and death induced by cell cycle reentry (as adult neurons are essentially post-mitotic cells). Additional potential death factors are metabolic imbalance, energy/oxygen alterations (hypo/hyperglycemia or hypoxia), accumulation of peroxynitrite, and oxygen free radicals.

Aberrant neuronal cell death is a major cause of acute and chronic neurodegenerative diseases [3,4]. Given the critical importance of neuronal death in the pathogenesis of many disorders, and considering that neurodegeneration is mediated by multiple causal mechanisms that may temporarily overlap, a deeper understanding of the types, mechanisms, and roles of neuroprotection is of fundamental importance to develop strategies to combat neurodegeneration [2,4,5]. Neuroprotection is broadly considered as a process that contributes to the salvage, recovery, or regeneration of the nervous system, its cells, structure, and function [4]. There are many neurochemical modulators in the nervous system that also exert neuroprotective effects. Among them, secretory neuropeptides are distributed widely throughout the central and peripheral nervous systems; they commonly act as complementary signals to “classic” neurotransmitters to fine-tune the neurotransmission, thereby controlling the balance between excitation and inhibition [6,7,8]. By definition, neuropeptides are small protein-like molecules (in some cases “normal” proteins), which function primarily as transmitter molecules in neuronal cells [8]. Some of them have been found to be important for the regulation of cell death/survival in different neuronal systems [9,10,11,12,13]. In particular, neuropeptides and their receptors are expressed widely in mammalian retinas, where they exert multifaceted functions both during development and in the mature animal [14].

Here, we focus our attention on the novel advances achieved in the last decade or so, with the aim of better understanding the neuroprotective roles of neuropeptides in the retina. In particular, the present paper: (i) provides information on the major neuropeptides/receptors involved in retinal disease, (ii) reviews recent results obtained in both in vitro and in vivo models on the mechanisms by which peptides may modulate retinal neuronal death/survival, (iii) gives indications on possible positive effects of neuroprotective peptides on retinal vascular lesions that occur in many pathologies, and (iv) emphasizes neuropeptide systems as potential targets for the treatment of retinal neurodegenerative diseases.

## 2. Angiotensin

The renin–angiotensin system (RAS) plays a major role in the regulation of blood pressure. Renin, a proteolytic enzyme derived from the precursor prorenin and primarily released by the kidneys, cleaves angiotensinogen to angiotensin I (AngI). AngI is further processed by angiotensin-converting enzyme (ACE) and ACE2 to different peptide cleavage products. Among them, angiotensin II (AngII) is the main effector of the RAS acting at the angiotensin type I and type 2 receptors (AT1R and AT2R) [15]. AngII also acts on the adrenal cortex, triggering the release of aldosterone, which binds to its receptor (mineralocorticoid receptor—MR) and contributes to electrolyte and water balance in the body [16].

A tissue-specific RAS has been identified in several organs [17]. In particular, a local RAS is present in the retina, where RAS components have been localized mainly to ganglion cells, but also to Müller cells, amacrine cells, bipolar cells, and photoreceptors. A local aldosterone system is likely to be expressed in the retina [18]. AngII, acting directly through AT1R or indirectly through induction of aldosterone release and activation of MR expressed in the retina, has been observed to induce reactive oxygen species (ROS) generation, production of advanced glycation end-products, inflammation, microglia activation, vascular leakage, neovascularization, Müller cell activation, and ganglion cell damage [18,19,20]. In addition, the retinas of transgenic (mRen2) 27 rats, which have high plasma prorenin levels, were characterized by increased apoptosis of inner neurons and photoreceptors, loss of capillaries, and increase of inflammatory cytokines [21]. Interestingly, in a rat model of preeclampsia (a disorder occurring during pregnancy and characterized by high blood pressure and retinal damage), systemic delivery of agonistic AT1R autoantibodies provoked histopathologic retinal changes, apoptosis of retinal cells, increased ROS formation, and reduction of electroretinogram (ERG) a- and b-waves [22]. Consistent with these data, an increase in the expression of prorenin, renin, AngII and AT1R has been reported in diabetic retinas [23] and increased levels of RAS components have been observed in the vitreous of patients with proliferative diabetic retinopathy (PDR) and macular edema [24,25]. On the other hand, AngI cleavage by ACE2 may produce Ang (1–7), which exerts anti-inflammatory and anti-angiogenic actions through its receptor, Mas. Similarly, AngII binding to AT2R may also antagonize the effects of AT1R activation [18]. Therefore, it appears that reduction of AngII expression (for instance, by blocking prorenin or renin actions) or blockade of AT1R, on the one hand, and stimulation of the ACE2/Ang (1–7)/Mas axis, on the other, may be exploited to counteract retinal damage occurring in retinal pathologies such as glaucoma, retinal ischemia, autoimmune uveitis, or diabetic retinopathy.

In in vitro experiments with purified rat retinal ganglion cells or cells of the 661W cell line, AT1R blockers such as telmisartan, valsartan, losartan, and candesartan were observed to prevent apoptosis and decrease ROS accumulation [26,27], while aliskiren (a renin inhibitor) prevented prorenin-induced expression of proinflammatory cytokines in cultured Müller cells [21]. In addition, irbesartan, another AT1R blocker, was reported to increase cell survival, improve ganglion cell dendritic arborizations, and reduce oxidative stress in cultured rat retinal explants [19]. These observations, indicating that inhibition of the prorenin/renin/AngI/AngII/AT1R pathway protects retinal cells from oxidative stress and apoptosis, were confirmed by observations in AT1R KO mice, in which retinal ganglion cell death induced by chronic alcohol consumption was significantly reduced with respect to wild-type animals [28].

Several investigations have provided evidence of a protective role exerted by AT1R inhibitors in in vivo models. For instance, systemic treatment with such inhibitors attenuated light-induced retinal damage in mice by reducing ROS accumulation, preventing photoreceptor apoptosis, and improving ERG responses [29]. Similarly, candesartan prevented retinal ganglion cell loss, thinning of the inner retina, and visual disturbances assessed with ERG in a retinal excitotoxicity mouse model [30]. Most of the studies have been conducted in rats or mice with increased intraocular pressure (IOP), used as models of glaucoma or ischemia–reperfusion, or in rats or mice with streptozotocin (STZ)-induced diabetes, used as models of diabetic retinopathy. Generally, in IOP models, AT1R blockade resulted in decreased ganglion cell loss, reduced ROS formation, and less extracellular glutamate [31,32,33,34]. Similar effects were induced by the renin inhibitor aliskiren, although the treatment did not seem to have any effect on retinal function as evaluated with ERG [35]. In diabetic models, blockers of AT1R, in addition to protecting the retina from oxidative stress, apoptotic cell death, and histopathologic damage [36,37,38], also prevented glial reaction, preserved mitochondrial integrity, increased the expression of neurotrophic factors, and improved functional ERG responses [36,37,39]. Finally, the renin inhibitor aliskiren was observed to prevent glial reaction, inflammation, and formation of acellular capillaries [21], while a prorenin receptor blocker inhibited inflammation and the diabetes-induced retinal expression of vascular endothelial growth factor (VEGF) [40].

A recent paper examined the expression of AngII and of Ang (1–7) in the retinas of normoglycemic and of diabetic rats. Both AngII and Ang (1–7) were localized to Müller cells. Interestingly, the diabetic condition resulted in increased AngII and decreased Ang (1–7) expression, while treatment of diabetic animals with the ACE inhibitor captopril reduced AngII and increased Ang (1–7) [41], indicating that, while the AngII-driven axis is associated to a pathologic condition, activation of the Ang(1–7)-driven axis is more compatible with a normal condition. In line with this view, studies have been conducted to investigate the possible therapeutic effects of ACE2 overexpression or of ACE2 activation in animal models of retinal disease. In experimental models of mouse autoimmune uveitis or of endotoxin-induced uveitis, the delivery of different formulations of ACE2 and/or Ang (1–7), as well as the administration of an ACE2 activator reduced retinal inflammation [42,43,44] and prevented histologic damage and functional deficits [43]. Similarly, both in rats with increased IOP and in rats with STZ-induced diabetic retinopathy, retinal ganglion cells were protected from apoptotic cell death by the administration of an ACE2 activator [45,46], while ACE2 gene delivery to diabetic mice or rats reduced oxidative stress, inflammation, vascular leakage, and the formation of acellular capillaries [47].

## 3. Glucagon-Like Peptide-1

Glucagon-like peptide-1 (GLP-1) is an incretin hormone secreted by the L-cells of the gastrointestinal tract in response to food. It stimulates glucose-dependent insulin secretion and inhibits the secretion of glucagon [48]. GLP-1 and its receptor GLP-1R are expressed in the brain, where GLP-1 may affect multiple neural circuits and modulate feeding behavior and reward [49]. Both GLP-1 and GLP-1R have been detected in human, rat, and mouse retinas at both the mRNA and the protein level [50,51,52,53,54]. Although it has been localized mainly to the ganglion cell layer, GLP-1R has also been reported in a rat Müller cell line [54], suggesting that this receptor may be expressed by Müller cells in mammalian retinas.

GLP-1R agonists such as exendin-4 (aka exenatide) or liraglutide were effective in protecting RGC-5 or R28 cells from damage caused by oxidative stress with a mechanism probably mediated by sirtuins [55,56]. Since oxidative stress is a causative event in many retinal pathologies, GLP-1R activation is likely to produce beneficial effects in a variety of conditions. For instance, intravitreal implants of beads with genetically modified cells producing GLP-1 decreased apoptosis and promoted survival of retinal ganglion cells in a rat model of optic nerve crush [57,58].

Perhaps the most extensive investigation of GLP-1 neuroprotective actions has been performed in models of diabetic retinopathy. That GLP-1 and GLP-1R are likely to play some role in human diabetic retinopathy is suggested by findings in human retinas reporting changes of GLP-1 or GLP-1R expression in the retinas of diabetic patients. In particular, one study conducted on diabetic patients with a diabetes duration of about six years reported a decrease of GLP-1 expression with respect to controls, but no changes were observed in GLP-1R expression [50]. In contrast, a study performed on the retinas of diabetic patients with a diabetes duration over 10 years, who had received laser photocoagulation and who were in an advanced stage of PDR, observed a decrease in GLP-1R expression with respect to the controls [51]. This discrepancy is likely due to the use of different techniques to detect GLP-1R or, more likely, to differences in the stage of diabetic retinopathy or in the treatment received by the patients. In the retinas of diabetic *db/db* mice, GLP-1 expression was decreased, while, similar to findings in diabetic patients without PDR, GLP-1R expression seemed to be unaffected by diabetes [50]. However, other studies in diabetic animal models reported downregulation of GLP-1R, which was prevented by administration of GLP-1 or of GLP-1R analogs [53,54,59,60]. Although somewhat contrasting, these observations indicate an involvement of GLP1/GLP-1R in the development of the disease.

The possible use of GLP-1, of GLP-1R analogs, or of inhibitors of dipeptidyl peptidase 4 (DPP4, the GLP-1 degrading enzyme) to treat diabetic retinopathy has been investigated in both in vitro and in vivo models. High glucose-induced apoptosis and mitochondrial changes in cells of the RGC-5 cell line were prevented by administrations of the GLP-1R agonist exendin-4 [61,62]. Similarly, exendin-4 also decreased the effects of high glucose in primary cultures of retinal Müller cells, where reduction of apoptosis and of glial fibrillary acidic protein (GFAP) expression was concomitant with inhibition of GLP-1R downregulation [59,60]. In diabetic animal models, treatments inducing increase of GLP-1R activation, including intravitreal, systemic, or topical administrations of GLP-1, of GLP-1R agonists, or of DPP4 inhibitors, commonly prevented cell loss and decrease of retinal thickness, reduced apoptosis, and activated prosurvival signaling pathways [50,52,53,54,55,60,63,64]. This increased resistance of retinal neurons to diabetic stress often resulted in significant functional improvement, as assessed by ERG [50,52,54,55,59,60,64], and in decreased glial activation, as indicated by reduced GFAP expression [50,60,64]. One possible mechanism by which GLP-1R activation protects the diabetic retina is likely to involve decreased accumulation of ROS and inhibition of oxidative stress [53,55,63], with the possible involvement of sirtuin1 and sirtuin3 [55]. Another mechanism may be related to an effect on glutamate excitotoxicity, as increased GLP-1R stimulation in the retinas of diabetic animals prevented both the downregulation of glutamate/aspartate transporter and the increase of retinal glutamate concentration [50,54,64]. Finally, neuronal protection may be induced by GLP-1 through an effect on inflammation. Indeed, linagliptin and exendin-4 have been reported to reduce the expression of pro-inflammatory factors in STZ rat retinas [63] and in a model of retinal ischemia–reperfusion [65], respectively.

Similar to other neuroprotective peptides, GLP-1 may also have an effect on VEGF expression. Indeed, exendin-4 has been observed to inhibit VEGF upregulation induced in vitro by high glucose or in the in vivo retina by a diabetic condition [59]. This anti-VEGF action is likely to result in decreased vascular lesions, as indicated by observations in diabetic or in ischemic retinas in which increased GLP-1R activation prevented blood–retinal barrier (BRB) breakdown, acellular capillaries, and pericyte loss [59,63,64,65].

## 4. Growth Hormone

Growth hormone (GH) is produced in the pituitary and has many documented effects throughout the body, particularly on cell differentiation, proliferation, and survival [66]. However, GH expression has also been found in tissues other than the pituitary. Indeed, the expression of genes or proteins related to GH has been reported in retinal ganglion cells of reptiles, birds, rodents, and primates, including humans [67,68,69], and low levels of GH in the human vitreous have been associated with retinal neurodegeneration [70]. In the retina, GH is expressed together with the GH receptor (GHR), suggesting a local autocrine/paracrine mode of action [71,72]. In addition, GH-releasing hormone (GHRH), which regulates the secretion of GH from the pituitary, is also expressed in the retina [68,73]. In primate retinas, expression of GHRH, GH, and GHR has been reported in all nuclear layers and in the retinal pigment epithelium (RPE) [68,69].

Although an excess of GH may alter visual function, as observed in ERG recordings from transgenic mice overexpressing bovine GH [74], the presence of a GH-related axis in the retina has been linked to pro-survival effects, mainly through the activation of anti-apoptotic pathways [75,76]. For instance, blockade of the GHRH receptor has been found to induce apoptotic cell death in a retinoblastoma cell line [77], and different studies in embryonic chick retinas or in immortalized avian retinal ganglion cells have provided evidence of the neuroprotective effects of GH against both the natural apoptotic death of retinal cells during development and the apoptosis of ganglion cells induced by retinal stress. In particular, knockdown of retinal GH in chick embryos resulted in increased apoptotic cell death [78], while other studies demonstrated the significant protective effects of GH against glutamate or kainate-induced retinal excitotoxicity [79,80]. In addition, GH may be effective at inducing some sort of neural regeneration, as GH administration was shown to protect retinal ganglion cell dendrites, promote synaptogenesis, and induce neurite outgrowth [80,81,82].

Similar to studies in chicks, GH protected retinal neurons from excitotoxic damage in the green iguana [72], while in the retinas of rats with STZ-induced diabetes GHRH agonists induced antioxidant and anti-inflammatory effects, thus promoting ganglion cell survival [73]. Finally, observations in postmortem human retinas reported that none of the ganglion cells expressing both GH and GRH immunoreactivity (about 35% of all the cells in the ganglion cell layer) was apoptotic, while other cells displaying TUNEL labeling did not express GH or GRH immunolabeling, suggesting that GH promotes survival in adult human retinal ganglion cells [76].

Regarding the mechanisms for the control of the retinal levels of GH, studies in immortalized quail retinal ganglion cells indicated that endogenous GHRH prevents cell death by increasing endogenous GH secretion [83], while other studies demonstrated that GH in the bloodstream translocates to the retina and internalizes into ganglion cells [84], suggesting that both exogenous (endocrine) and local (paracrine/autocrine) mechanisms may be involved in the regulation of retinal GH. Regarding the possible mechanisms mediating the protective effects of GH on retinal neurons, studies in embryonic neuroretinal cells reported that GH overexpression or GH administration may induce expression of brain-derived neurotrophic factor and of neurotrophin 3 [80], indicating that GH protective actions may be mediated by these neurotrophins. However, most data indicate that neuroprotective actions of GH are mediated in large part by another neurotrophic factor, namely insulin-like growth factor-1 (IGF-1) both in the developing retina [78,79,85] and in retinas under stress conditions [72]. Indeed, IGF-1 is the major mediator of growth hormone activity in humans and the IGF-I/IGF-IR system has been found to be expressed in the retina [86].

## 5. Neuropeptide Y

Neuropeptide Y (NPY) is involved in various physiological and homeostatic processes in both the central and peripheral nervous systems. NPY has been identified as the most abundant peptide present in the mammalian central nervous system. As excellently reviewed by Santos-Carvalho and colleagues [87,88], NPY is expressed and functionally active in different retinal cells of non-mammalian and mammalian species, where it can have paracrine or autocrine effects by acting on NPY receptors. The NPY receptors are expressed in different retinal cell types, such as RPE, photoreceptors, horizontal, amacrine and ganglion cells, Müller cells, and microglia.

NPY exerted a neuroprotective effect against toxicity (necrosis and apoptosis) induced by MDMA (methylenedioxymethamphetamine, often known as “ecstasy”) in rat retinal mixed cell cultures containing neurons, astrocytes, Muller cells, and microglial cells [89]. In rat retinal neurons, NPY inhibited the increase in intracellular Ca^2+^ evoked by KCl through the activation of NPY Y_1_, Y_4_, and Y_5_ receptor subtypes, likely contributing to its neuroprotective effect [90]. Accordingly, in recent years, studies have suggested that the NPY system could be exploited for potential protective strategies in retinal degenerative diseases [87,88].

The induction of diabetes in rats (as in STZ-treated animals) decreased the retinal NPY mRNA levels, as well as the protein levels of NPY and of NPY Y_5_ receptor [91]. Of interest, NPY was demonstrated a neuroprotective agent against necrotic and apoptotic cell death induced by cytotoxic glutamate in rat retinal cells both in vitro and in vivo. In particular, NPY protected retinal cells against glutamate-induced necrosis by activating NPY Y_2_, Y_4_, and Y_5_ receptors and from apoptosis by activating NPY Y_5_ receptors [92]. More recently, NPY attenuated the increase of intracellular Ca^2+^ triggered by glutamate in purified retinal ganglion cells and in ex vivo rat retinal preparations, mainly via NPY Y_1_ receptor activation [93]. The NPY Y_1_ receptor activation was also able to modulate directly ganglion cell responses by attenuating the NMDA-induced increase in ganglion cell spiking activity. NPY pretreatment also prevented NMDA-induced cell death, although in a rat model of retinal ischemia–reperfusion injury pretreatment with NPY could not prevent apoptosis or rescue retinal ganglion cells and retinal function [93], thus introducing some doubts about NPY’s translational potential. In this line, a worsening effect induced in vivo by NPY following an ischemic insult has been reported. In particular, intravitreal injection of NPY after ischemia induction in pigs caused a significant reduction of retinal function, as evaluated by standard and global-flash multifocal ERG. This reduction was accompanied by histological damage, as for instance the reduction of ganglion cells, likely via NPY Y_1_ and Y_2_, but not Y_5_ receptors [94]. 

## 6. Opioid Peptides

Opioid peptides are known as powerful analgesics, but they are involved in a variety of functions in the organism. Their effects are mediated by δ, κ, and µ opioid receptor subtypes [95]. Opioid receptors have been detected in virtually all major organ systems. In particular, the presence of functional opioid receptors has been reported in the retina, optic nerve, and optic nerve head astrocytes [96,97].

Evidence has been provided that the administration of morphine, a broad-range opioid agonist, is effective in reducing ischemic retinal injury [96]. Regarding specific opioid receptor subtypes, the δ opioid receptors have been implicated in neuroprotective effects in the retina [98]. The neuroprotective effects of opioids have been studied mainly in models of retinal ischemia, and they have been reviewed previously [99]. Here, we provide an update of the most recent findings.

In a rat model of ischemia–reperfusion injury caused by elevated IOP, morphine inhibited the production of the proinflammatory cytokine tumor necrosis factor α (TNFα), an effect antagonized by naloxone, a nonselective antagonist of opioid receptors [97]. In addition, a naloxone-sensitive effect of morphine was also reported in glaucomatous rats, where opioid agonism was observed to decrease ganglion cell death, to inhibit TNFα, caspase-8, and caspase-3 expression, and to improve functional retinal responses, as evaluated with pattern ERG [100]. Similar findings were reported in glaucomatous rats treated with a δ opioid receptor agonist [101]. The neuroprotective effect of δ agonism may be mediated, at least in part, by inhibition of inducible nitric oxide (NO) synthase (iNOS). Indeed, NO, mainly produced by iNOS, may play a detrimental role in glaucoma, and its inhibition by a δ opioid agonist results in neuroprotection [102]. The neuroprotective effects consequent to δ opioid activation are likely to be mediated through the PI3K/Akt pathway [103]. Activation of δ opioid receptors may induce ameliorative changes other than direct neuroprotection in models of retinal injury. Indeed, it has been shown that ARPE-19 cells challenged with high glucose decrease TNFα production and preserve tight junction proteins when treated with epicatechin, which acts as a δ opioid activator, thus indicating a protective effect of opioid peptides on the integrity of the outer BRB [104]. Although most studies on the retinoprotective effects of opioid peptides are concerned with ischemic models and with the δ receptor subtype, a recent study using a model of retinal excitotoxicity and administrations of the opioid peptide ß-endorphin (a ligand of the µ opioid receptor) suggests that not only the δ but also the µ subtype of the opioid receptors may play important neuroprotective functions in retinal disease [105].

The data reported above indicate protective effects of opioid receptors that may be antagonized by the opioid antagonist naloxone, thus indicating naloxone as a detrimental compound when the objective is retinal neuroprotection. However, the neuroprotective effects of naloxone have been demonstrated in the central nervous system [106], although these effects are unlikely to depend on inhibition of the opioid system [107,108]. In the retina, naloxone was reported to protect from light-induced photoreceptor degeneration through the inhibition of activated microglia [109]. In a mouse model of age-related macular degeneration, naloxone has been shown to reduce the progress of retinal lesions, the production of pro-inflammatory cytokines, and microglia aggregation [110]. Since naloxone’s greatest affinity is for the µ opioid receptor and the µ3 receptor is linked to NO production [111], naloxone may protect the retina from NO-induced neuronal damage playing an inhibitory action at this opioid receptor.

## 7. Somatostatin

Somatostatin (somatotropin release inhibiting factor—SRIF) is considered to be one of the key physiologically active neuropeptides expressed in the retina [112,113]. Five SRIF receptor subtypes coupled to different G-proteins have been cloned, namely sst1–5 [114], and they modulate the actions of multiple second messengers/transduction pathways [114,115,116,117,118]. SRIF receptors have been detected in different areas of the central nervous system [114,119,120], including the retina, where sst_1_ and sst_2_ are the most widely expressed in multiple retinal layers and cell types [112,113,121].

Clinically, the fact that lower vitreous levels and lower intraocular production of SRIF were found in patients with diabetic macular edema, chronic uveitis macular edema, and quiescent intraocular inflammation [122,123] suggests that SRIF alterations may be directly involved in the pathogenesis of these conditions. On the other hand, different pre-clinical observations supported a role for SRIF as a neuroprotective factor in a variety of retinal diseases [13,112,124,125]. The importance of the SRIF system in protecting the retina from noxious stimuli has been confirmed in recent years. 

In the retinas of diabetic rats, treatment with SRIF eye drops inhibited glutamate accumulation and glutamate/aspartate transporter downregulation [126]. SRIF administration also prevented ERG abnormalities, glial activation, apoptosis, and the misbalance between proapoptotic and survival signaling [126]. Using in vitro systems mimicking diabetic-like conditions, SRIF was demonstrated to decrease endothelial cell apoptosis without affecting the response of human retinal pericytes expressing sst1 [127]. On the other hand, SRIF reduced the expression of pro-inflammatory markers and counteracted the imbalance between apoptotic and survival intermediates in human retinal pericytes exposed to conditioned media from activated microglia [128], thus suggesting a possible anti-inflammatory role in the early phases of PDR, a disease in which neurodegeneration is thought to occur prior to microvascular alterations. In this respect, SRIF and octreotide, a sst_2_-preferring agonist, reduced apoptosis as well as VEGF expression and release in retinal explants exposed to stressors similar to those characterizing diabetic retinopathy, that is, high glucose, oxidative stress, or advanced glycation end-products [129,130]. SRIF was also shown to reduce high glucose-induced apoptosis in photoreceptor cells [130].

Retinal VEGF patterns are affected profoundly by the onset of an ischemic state and may represent a fast response of the VEGF system to severe shortage of nutrients and oxygen in retinal neurons [131]. It should be noted that an ischemic condition not only causes cell death, but also induces a vascular response and is a common clinical entity since it causes visual impairment and blindness [132,133]. There are indications that endogenous SRIF mediates the retinal protective effects exerted by anti-inflammatory and neuroprotective factors during ischemic injury [124]. Notably, the activation of SRIF receptors, likely sst_2_, protected neurons from apoptosis by ischemic damage and reduced VEGF overexpression as well as glutamate release [131,134,135,136,137,138]. These effects are likely to be due, at least in part, to a reduction of VEGF release by damaged neurons and its accumulation in the retinal capillaries [131]. Different approaches also demonstrated that octreotide treatment counteracts ischemia-induced oxidative stress and modulates various metabolic responses during ischemic damage [136,137]. Of interest, recent data indicated a cross-talk between apoptosis and autophagy in the ischemic or hypoxic retina [139,140]. This cross-talk may be altered by stressing conditions favoring apoptosis, but it may be re-equilibrated by autophagy-stimulating substances. In particular, the reported antiapoptotic actions of octreotide seem to be, at least in part, the result of a stimulation of the autophagic flux [139].

## 8. Substance P

The peptides of the tachykinin family are characterized by a common C-terminal amino acid sequence (Phe–X–Gly–Leu–Met–NH2). Substance P (SP) is the best characterized neuropeptide of this family, but other tachykinins have also been described so far, the two main ones being neurokinin A (NKA) and neurokinin B (NKB) [141]. The tachykinins act thorough specific NK receptors, namely NK1R, NK2R and NK3R. SP, NKA, and NKB have the highest affinity for NK1R, NK2R, and NK3R, respectively, but they do not bind them in a selective manner [141].

In the retina, SP is highly expressed in ganglion cells and in the inner plexiform layer where it has been localized in sparse amacrine cells, and a similar localization pattern has been described for the other two tachykinins, NKA and NKB [14,142,143]. There is evidence highlighting the potential for endogenous SP as a treatment for retinal damage. For instance, a greater content of SP has been reported in the retina in response to acute stress or in retinal pathologies [144,145]. In addition, results from studies in diabetic rats showed that the levels of SP in the retina and serum were reduced, with an associated increase in apoptosis and caspase-3 activity, while the restoration of endogenous SP levels paralleled the inhibition of apoptosis [146]. Similarly, the levels of SP and NKA/NKB decreased in an NMDA–excitotoxicity model of the rat retina [147], while both the severe retinal destruction and the dense neovascularization in laser-induced retinal degeneration models recovered after SP administrations [148]. Noteworthy, SP treatment suppressed early inflammatory responses in proliferative vitreoretinopathy-like retinal damage, with inhibition of cell death, limitation of the appearance of fibroblastic cells, and delay of the progression of retinal degeneration [149].

In ischemic retinas, SP reduced apoptotic cell death, VEGF overexpression, and glutamate release, also counteracting the oxidative stress and perturbations in the metabolome induced by the ischemic insult [136]. The NK1R were identified as the receptor subtype possibly involved in SP protective mechanisms [105,150]. Recently, the protective effect of SP against NMDA excitotoxic apoptosis of ganglion cells was established in ex vivo retinal explants and in vivo murine models [151]. Of interest, SP was also found to maintain endothelial tight junctions and to decrease VEGF-induced vascular permeability, thus inhibiting VEGF-induced BRB breakdown [151]. Finally, SP was effective at protecting RPE cells from oxidative stress-induced cell death via the NK1R [152] and in ameliorating RPE epithelial–mesenchymal transition and fibrotic change after inflammatory stimuli [149].

## 9. Vasoactive Intestinal Peptide and Pituitary Adenylate Cyclase Activating Polypeptide

Vasoactive intestinal peptide (VIP) and pituitary adenylate cyclase-activating polypeptide (PACAP) belong to a peptide superfamily that also includes secretin and glucagon. Their receptors are G protein-coupled receptors that can be classified into two groups: PAC1R, which binds PACAP with higher affinity than VIP, and VPAC receptors (VPAC1R and VPAC2R), which bind PACAP and VIP with similar affinities [153]. VIP has been observed to support neuronal survival in both physiological and pathological conditions in the central as well as in the peripheral nervous system [154]. Similarly, widespread neuroprotective properties of PACAP, mediated mainly by the PAC1R receptor, have been reported in a variety of in vitro and in vivo models and have been extensively reviewed [11,12,155,156,157,158,159,160,161,162,163,164,165].

Both VIP and PACAP occur in the retina. In particular, VIP has been reported in a population of amacrine cells, which, in the mouse retina, is likely to include different cell types [166]. PACAP, instead, localized to horizontal, amacrine, and ganglion cells [167,168]. The PACAP-containing ganglion cells have been identified as the melanopsin-expressing ganglion cells originating the retinohypothalamic tract, which connects the retina with the suprachiasmatic nucleus of the hypothalamus and is involved in the regulation of biological rhythms [169]. PAC1R were observed in amacrine and ganglion cells [170,171] as well as in in rat primary cultures of Müller cells [172]. Functionally, both VIP and PACAP have been found to be implicated in retinal development [14] (see also [173] for references) and to be involved in information processing of visual stimuli [174,175,176]. In addition, PACAP interacts with glutamate in the transmission of light stimuli to the suprachiasmatic nucleus [177].

### 9.1. Neuroprotective Effects of VIP in the Retina

VIP has been reported to protect retinal ganglion cells against glutamate excitotoxicity in vitro [178] and to reduce the retinal neurodegenerative effect of ischemia–reperfusion injury through an antioxidant action [179]. VIP may also mediate the reduction of t e inflammatory response and the improvement of retinal function induced by vagal stimulation in rats with acute ocular hypertension [180]. Recently, both PACAP and VIP have been shown to efficiently attenuate ischemic retinal degeneration induced by bilateral common carotid artery occlusion (BCCAO) when are bound to the cell penetrating peptide TAT and administered through eye drops [181]. However, VIP has been shown to be 10 times less active than PACAP in ischemic retinopathy [182]. In a retinal disease such as diabetic retinopathy, VIP may contribute to the protection of retinal neurons by reducing outer BRB dysfunction [183,184], probably through an inhibition of VEGF and of hypoxia-inducible factor 1α (HIF1-α), the main transcriptional regulator of VEGF expression [185].

The neuroprotective effects of VIP may be either direct through activation of the PAC1R [181], or indirect through regulation of activity-dependent neurotrophic protein (ADNP) [186,187,188]. Indeed, both ADNP and an 8-amino acid peptide derived from ADNP (referred to as NAP) display important neuroprotective activities [189]. In particular, NAP has been reported to enhance both survival and neurite outgrowth in retinal ganglion cells in vitro [190], while intraocular or intraperitoneal NAP administrations resulted in significant protection of retinal ganglion cells after retinal ischemia or optic nerve crush [191]. Similarly, intraocular or intravenous injections of NAP protected against laser-induced retinal damage [192]. In addition, stable transfection of NAP into retinal Müller cells with constant NAP production protected both Müller cells and retinal neurons from damage induced by hypoxia [193].

Interestingly, NAP, similar to VIP, seems to play different protective effects against pathologic changes induced by diabetic retinopathy, as it reduced both inflammation [194] and apoptosis [195] as well as the levels of HIF1-α and of VEGF [196] in retinas of rats with STZ-induced diabetes, and protected the integrity of the outer BRB exposed to hyperglycemic/hypoxic or inflammatory insult [194,197].

### 9.2. Neuroprotective Effects of PACAP in the Retina

The retinoprotective effects of PACAP have been widely investigated and the results of these studies have been reviewed previously [198,199,200]. Here we provide a summary of the findings of the last few years.

Recent evidence indicates that physiological expression levels of PACAP in the retina are necessary to maintain retinal integrity. Indeed, retinas of PACAP KO mice were characterized by abnormal sprouting of horizontal and rod bipolar cell dendrites, decreased ganglion cell number, altered MAPK signaling pathway, and GFAP upregulation in Müller cells [201], which is known to appear in response to retinal injury [202]. In addition, PACAP KO retinas displayed significantly worse structural and functional damage with respect to wild types following lipopolysaccharide-induced eye inflammation [203], confirming that PACAP expression in the retina may represent a natural defense against injury. Consistent with this hypothesis, upregulation of both PACAP and PAC1R has been reported in retinas of rats after optic nerve crush [204].

Intravitreal administrations of PACAP have been shown to inhibit apoptosis and promote survival of retinal ganglion cells in different models of retinal injury [205,206]. Since PACAP is subjected to rapid enzymatic hydrolysis in the extracellular environment [207], the efficacy of a more stable, cyclized form of PACAP has been tested both in vitro and in vivo. In these studies, RGC-5 cells exposed to ultraviolet irradiation showed decreased apoptosis and less ROS generation when treated with cyclic PACAP, while intravitreal injection of the compound enhanced ERG and ganglion cell survival in rat retinas exposed to excitotoxic injury [208].

PACAP has been found to be very effective in protecting the retina from ischemia. In an ex vivo model, PACAP decreased apoptosis and glutamate accumulation, reduced peroxidized lipids and inflammatory mediators, and induced normalization of glutathione homeostasis. In addition, PACAP decreased VEGF expression, which was observed to increase in the ischemic retina [136]. In the BCCAO model, intravitreal PACAP was observed to ameliorate ERG responses [209], while intravitreal administrations of maxadilan (a PAC1R agonist) dose-dependently reduced the thinning of retinal layers and the loss of cells in the ganglion cell layer [210]. The ischemic damage was also combated by topical administrations of different formulations of PACAP through eye drops. Similar to intravitreal delivery, PACAP eye drops protected the retina from thinning and from cell loss [181,211,212], while they also reduced GFAP upregulation in Müller cells [211,212]. 

Some studies have investigated possible effects of PACAP against retinal damage caused by diabetic retinopathy. Both PACAP administration to ex vivo retinal explants treated with diabetic stressors and PACAP intraocular delivery in rats with STZ-induced diabetes protected the retina from apoptosis [129,213], maintained retinal synaptic integrity [214], and prevented the expression of inflammatory cytokines [215]. Interestingly, PACAP protective and antiapoptotic effects were paralleled by inhibition of upregulation of HIF1-α, of VEGF, and of VEGF receptors (VEGFRs) in retinal explants [129], in STZ rats [215,216], and in pigment epithelial cells of the ARPE-19 cell line [184].

## 10. Other Peptides

In addition to the neuropeptides discussed above, a variety of other peptidergic molecules with documented retinoprotective properties have been found, although available data in the literature are far from abundant. Here, we provide a (probably incomplete) summary of them.

### 10.1. α-Melanocyte-Stimulating Hormone 

α-Melanocyte-stimulating hormone (α-MSH) is a widely-distributed 13-amino acid peptide derived from proteolytic cleavage of proopiomelanocortin [217]. It acts at 5 subtypes of G protein-coupled receptors (melanocortin receptors, MC1R to MC5R) [218] and regulates a variety of physiological functions, ranging from thermoregulation [219] to metabolism [220]. α-MSH is known to protect against ischemic damage of the brain [221]. In the retina, α-MSH acting at MC4R protected developing chicken retinas from glutamate induced excitotoxicity [222]. In addition, α-MSH protected the rat retina from both functional and structural damage induced by ischemia–reperfusion [223], suppressed inflammation and maintained the retinal structure in a mouse model of experimental autoimmune uveitis [224], and protected photoreceptors from degeneration in a rat model of retinal dystrophy [225]. In addition, intravitreal injections of α-MSH protected both the neuroretina and retinal vessels from oxidative stress and cell death in a rat model of STZ-induced diabetes [226]. Finally, α-MSH inhibited BRB breakdown and vascular leakage, improving both functional and morphological characteristics in early diabetic retinas, likely acting via MC4R [227].

### 10.2. Apelin

Apelin is an endogenous oligopeptide ligand for the G protein-coupled receptor APJ [228] and it has been reported to exert neuroprotective actions in the central nervous system (see [229] for references). In the retina, apelin has been reported in Müller cells [230,231], while APJ receptors have been localized to ganglion cells and to cholinergic amacrine cells [229]. Exogenous apelin prevented Müller cell apoptosis and stimulated Müller cell viability and migration under normal, hypoxic, or glucose-free conditions [230,231], while in an in vivo mouse model of retinal excitotoxicity, apelin was found to protect retinal ganglion cells from apoptosis and to ameliorate functional retinal responses, [229,232]. The protective effect of apelin against retinal excitotoxic damage was reported to be mediated by [229] or to be independent from [232] activation of APJ receptors.

### 10.3. Bradykinin

Bradykinin is a component of the kallikrein–kinin system, which may have a role in the development of diabetic retinopathy [233,234]. Kinins are important inflammatory mediators and exert their effects by binding two G-protein coupled bradykinin receptors named B1R and B2R [235]. Most components of the kallikrein-kinin system have been identified in the retina (see [236] for references). In particular, the B1R is overexpressed in the retina of rats with STZ-induced diabetes, where it is involved in BRB breakdown [237,238] suggesting a detrimental role of B1R in the development and the progression of diabetic retinopathy. Indeed, administrations of B1R blockers to STZ rats reduced retinal plasma extravasation, leukostasis, ROS formation, and mRNA levels of inflammatory mediators and of VEGFR2, and restored retinal Na^+^/K^+^-ATPase activity [236,239]. In addition, intravitreal injections of bradykinin in rats increased BRB permeability, an effect prevented by a B2R antagonist [240]. In apparent contrast with these findings, a recent investigation using two different models of diabetic mice reported that pancreatic kallikrein may activate B1R and B2R and ameliorate retinal oxidative stress, inflammation, apoptosis, acellular capillary formation, and vascular leakage [241].

### 10.4. Calcitonin Gene-Related Peptide

Calcitonin gene-related peptide (CGRP) and its receptors have been detected in the rat retina [242]. A protective role of endogenous retinal CGRP was suggested by studies in rats, in which a CGRP receptor antagonist was demonstrated to worsen the apoptotic rate of retinal ganglion cells following ischemia caused by acute myocardial infarction [243]. In addition, capsaicin-induced CGRP upregulation effectively protected retinal ganglion cells from apoptosis in retinas of rats with STZ-induced diabetes [244] or in rat retinas challenged with an excitotoxic insult [105,150].

### 10.5. Ghrelin

Ghrelin is a peptide hormone secreted by the stomach that is involved in regulation of food intake and energy balance. It acts at its receptor GH secretagogue receptor type 1a (GHSR-1a) [245]. In patients with glaucoma, ghrelin levels in the anterior chamber were reported to be significantly lower than in controls [246]. In rats with experimental glaucoma, ghrelin was effective in reducing autophagy and glial reaction and in protecting the retinal cells from oxidative stress and apoptosis [247,248], while ghrelin activation of GHSR-1a significantly protected RGC-5 cells from rotenone-induced toxicity [249]. Finally, obestatin, a peptide encoded by the ghrelin gene, has been reported recently to protect RGC-5 cells from oxidative stress by activating the TrkB pathway with a mechanism that is likely to involve GLP-1R [250].

### 10.6. Insulin

The insulin receptor is widely expressed in the neural retina and in the RPE [251]. Although there is some evidence indicating a neuroprotective role of insulin in the retina, it has not been investigated in detail. In rats with STZ induced diabetes, insulin has been reported to protect significantly retinal function, as assessed with ERG, and reduce retinal cell apoptosis, glial activation, VEGF upregulation, and BRB damage [252]. In addition, insulin receptors expressed by the RPE were reported to support photoreceptors in the diabetic retina [253]. A recent review suggests that, more than insulin, the prohormone proinsulin is likely to exert significant neuroprotective actions in the retina [254].

### 10.7. Prolactin

Prolactin is another peptide hormone whose receptors have been identified in the retina [255]. Similar to insulin, there are also reports suggesting a neuroprotective action of this hormone in the retina, although the evidence is quite limited. Prolactin is likely to exert antioxidant actions in the retina [256]. In a model of light-induced retinal degeneration, experimentally induced hyperprolactinemia limited photoreceptor apoptosis, gliosis, and changes in neurotrophin expression, and it preserved the ERG responses [257]. In addition, vasoinhibins, a family of peptides originating from the proteolysis of prolactin [258], prevented the excessive vasopermeability associated with diabetes [259], decreased bradykinin-induced BRB permeability, and reduced the levels oxidative stress in retinas of STZ rats [240].

### 10.8. Urocortin

Urocortin 2 (Ucn 2) is a corticotropin-releasing factor (CRF) paralog preferentially activating CRF2 receptors [260], which have been identified in the retina [261]. Intraocular administrations of Ucn 2 have been reported to preserve retinal thickness and promote ganglion cell survival in the rat BCCAO model [262], while in a model of excitotoxicity-induced retinal degeneration, Ucn 2 has been observed to rescue neurochemically-identified bipolar and amacrine cells [263].

## 11. Concluding Remarks

Neuropeptide expression in vertebrate retinas has been known for many years and neuropeptide functions have been found to involve neuromodulation and participation to visual information processing within the retina. More recently, an increasing number of peptidergic substances expressed in the retina together with their receptors have been recognized to play neuroprotective actions in a variety of models of retinal disease. Generally, neuropeptides are likely to exert antioxidant or anti-inflammatory actions, or they may limit extracellular glutamate, thereby promoting retinal neuronal survival. It is interesting to note that in many circumstances neuroprotective effects of neuropeptides have been described together with a positive effect against the vascular lesions characterizing some retinal diseases, such as, for instance, diabetic retinopathy. These observations indicate a link between neural and vascular damage in these diseases and indicate that peptide neuroprotection may also prevent pathologic vascular changes. Additionally, there is a general agreement that neuropeptides are coupled to multiple components of transduction pathways [13,264], which may converge in restoring neuronal functions. The diversity of signaling reflects the pleiotropic actions of peptides; at the cellular level, many mechanisms are involved in the amplification effects. Given that different neuropeptides are associated with beneficial effects against retinal neurodegeneration, it would also be of interest to explore potential common mechanisms at the second messenger level in relation to the actions of marketed drugs, as previously suggested for antidepressants [265]. 

However, the fact that the investigation of possible peptide-based therapeutic approaches is still at the preclinical level, with only a few exceptions, indicates that there are problems in the development of such strategies. One of these problems is represented by the low bioavailability of peptidergic substances, due to their rapid degradation in the extracellular environment. Thus, the challenge is to find new peptide analogs or peptide receptor agonists with higher resistance to degrading enzymes and peptide formulations affording better pharmacokinetics and bioavailability. New peptide formulations should also allow easier delivery to the retina, avoiding the need of invasive intraocular injections in favor of methods for oral or topical delivery. A significant advancement in this field could come from the conjugation of neuropeptides or peptide analogs with different types of nanoparticles [266]. In conclusion, the use of peptidergic neuroprotectants may lend unforeseen value and these substances may be regarded as a powerful tool for the development of therapies to cure neurodegenerative as well as vascular retinal diseases.

## Abbreviations

ACE	angiotensin-converting enzyme
ADNP	activity-dependent neurotrophic protein
AngI	angiotensin I
APJ	apelin receptor
AT1R and AT2R	angiotensin type I and type 2 receptors
B1R and B2R	bradykinin receptors 1 and 2
BCCAO	bilateral common carotid artery occlusion
BRB	blood–retinal barrier
CGRP	calcitonin gene-related peptide receptor
CRF	corticotropin-releasing factor
DPP4	dipeptidyl peptidase 4
ERG	electroretinogram
GFAP	glial fibrillary acidic protein
GH	growth hormone
GHR	GH receptor
GHRH	GH-releasing hormone
GHSR-1a	GH secretagogue receptor type 1a
GLP-1	glucagon-like peptide-1
GLP-1R	GLP-1 receptor
HIF1-α	hypoxia-inducible factor 1α
IGF-1	insulin-like growth factor-1
iNOS	inducible NO synthase
IOP	intraocular pressure
MDMA	methylenedioxymethamphetamine
MC1-5R	melanocortin receptors 1-5
MR	mineralocorticoid receptor
NAP	8-amino acid peptide derived from ADNP
NK1-3R	NK receptors 1-3
NKA and NKB	neurokinin A and B
NO	nitric oxide
NPY	neuropeptide Y
PAC1R	PACAP receptor 1
PACAP	pituitary adenylate cyclase-activating polypeptide
PDR	proliferative diabetic retinopathy
RAS	renin–angiotensin system
ROS	reactive oxygen species
RPE	retinal pigment epithelium
SP	substance P
SRIF	somatotropin release inhibiting factor—somatostatin
sst1–5	SRIF receptors 1-5
STZ	streptozotocin
TNFα	tumor necrosis factor α
Ucn 2	urocortin 2
VEGF	vascular endothelial growth factor
VEGFRs	VEGF receptors
VIP	vasoactive intestinal peptide
VPAC1R and VPAC2R	VIP and PACAP receptors 1 and 2
α-MSH	α-melanocyte-stimulating hormone
